# The assessment of Twitter discourse on the new COVID-19 variant, XBB.1.5, through social network analysis

**DOI:** 10.1016/j.jvacx.2023.100322

**Published:** 2023-06-07

**Authors:** Ikhwan Yuda Kusuma, Hening Pratiwi, Shafa Fitri Khairunnisa, Dian Ayu Eka Pitaloka, Arie Arizandi Kurnianto

**Affiliations:** a Pharmacy Study Program, Faculty of Health, Universitas Harapan Bangsa, Purwokerto 53182, Indonesia; b Department of Pharmacy, Faculty of Health Sciences, Jenderal Soedirman University, Purwokerto 53122, Indonesia; c Center of Excellence in Higher Education for Pharmaceutical Care Innovation, Universitas Padjadjaran, Sumedang 45363, Indonesia; d Department of Pharmacology and Clinical Pharmacy, Faculty of Pharmacy, Universitas Padjadjaran, Sumedang 45363, Indonesia; e Doctoral School of Health Sciences, Faculty of Health Sciences, University of Pécs, Pécs, Hungary

**Keywords:** Covid-19, XBB.1.5, Variant, Social network, Twitter, Sentiments

## Abstract

**Background:**

XBB.1.5 is a new subvariant of the SARS-CoV-2 Omicron variant with increased transmissibility and immune escape potential. Twitter has been used to share information and assess this subvariant.

**Objectives:**

This study aims to investigate the channel graph, key influencers, top sources, most trends, and pattern discussion, as well as sentiment measures related to Covid-19 XBB.1.5 variant, by using social network analysis (SNA).

**Methods:**

This experiment involved the collection of Twitter data through the keywords, “XBB.1.5″, and NodeXL, with the obtained information subsequently cleaned to remove duplication and irrelevant tweets. SNA was also performed by using analytical metrics to identify influential users and understand the patterns of connectivity among those discussing XBB.1.5. on Twitter. Moreover, the results were visualized through Gephi software, with sentiment analysis performed by using Azure Machine Learning to categorize tweets into three categories, namely positive, negative, and neutral.

**Results:**

A total of 43,394 XBB.1.5-based tweets were identified, with five key users observed with the highest betweenness centrality score (BCS), namely “ojimakohei”(red), mikito_777 (blue), “nagunagumomo” (green), “erictopol” (orange), w2skwn3 (yellow). The other hand, the in-degree, out-degree, betweenness, closeness, and eigenvector centrality scores of the top 10 Twitter users to explain various patterns and trends and “ojimakohei” was highly central in the network. Most of the top domains (sources) used in XBB.1.5 discourse originated from Twitter, Japanese websites (co.jp and or.jp), and scientific analysis links (biorxiv.org and cdc.gov). This analysis indicated that most of the tweets (61.35 %) were positively classified, accompanied by neutral (22.44 %) and negative (16.20 %) sentiments.

**Conclusion:**

Japan was actively engaged in evaluating the XBB.1.5 variant, with influential users playing a crucial role. The preference for sharing verified sources and the positive sentiment demonstrated a commitment to health awareness. We recommend fostering collaborations between health organizations, the government, and Twitter influencers to address COVID-19-related misinformation and its variants.

## Introduction

The Omicron variant of SARS-CoV-2 gave rise to a new subvariant called XBB.1.5 in early 2023 [1]. This subvariant is a recombinant of two BA.2 sub lineages and a subgroup of the XBB variant, with an extra spike receptor-binding domain (RBD) alteration, S486P [2]. According to the World Health Organization, XBB.1.5 was prevalent in 38 nations, with the UK Health Security Agency (UKHSA) stating that the variant was most likely to be highly dominant in the United Kingdom despite the country containing fewer than 5 % of all SARS-CoV-2 samples sequenced in the final week of 2022. The subvariant variation combines immune escape characteristics with a heightened ACE-2 binding affinity, potentially leading to increased transmissibility. Although XBB.1.5 is capable of elevating incidence after the present wave, the establishment of the trajectory is still very early. Based on the Centers for Disease Control and Prevention, the variation was “growing swiftly” in the US and was considered responsible for about 28 % of the present cases (week ending 7 January 2023) [3].

Several scientists, public health officials, and social members have also raised concern and discussion about XBB 1.5, as well as the efficacy of the available vaccines [4]. Although the variation has not been extensively studied, it still obtained various attention, specifically on social media platforms such as Twitter. From this context, Twitter is a widely used social media platform that allows users to share short messages, known as tweets, with a large audience. Regarding its real-time nature and large user base, the platform has become a valuable source of information and discussions on various topics, including public health [5], [6]. Twitter has also been frequently used to share information on COVID-19 breakthroughs throughout the pandemic period [7].

Based on several previous studies, various limitations were observed in the analysis of COVID-19 information sharing on Twitter. This indicated a majority of unreliable or less reliable information sources, for instance, in the pandemic Twitter networks [8], [9]. From this context, the performance of comprehensive Social Network Analysis (SNA) was encouraged regarding XBB 1.5. This is because SNA is an approach examining the social dynamics and structure of groups, organizations, and communities [10]. Several studies have employed this analytical approach to explore the conversations related to many diseases, such as COVID-19 [11], [12], [13] melanoma [14], myeloproliferative neoplasms [15], brain tumor [16], measles [17], Zika [18], Flu [19] and Ebola [18]. SNA also provides a potent lens to comprehend the social interactions, relationships, and influences moulding health-related behaviours and results, specifically examining the XBB 1.5 issue on Twitter users. Therefore, this study aims to investigate the methods by which individuals use media platforms to share their experiences while discovering important public influencers and topics related to XBB 1.5 through social network analysis (SNA). From this report, the following study questions are observed,

(1) Which social network structures and dynamics are most relevant to the XBB1.5 discussion on social media?
(2) Who are the key social influencers in the XBB 1.5 discussion on social media?
(3) What are the most frequent issues raised in discussions about XBB 1.5 on social media?
(4) What are the sentiments expressed in social media conversations related to XBB 1.5?

The introduction provides a comprehensive overview of the XBB.1.5 subvariant, its origin, prevalence, and potential implications. It highlights the combination of immune escape qualities and a heightened ACE-2 binding affinity, which may contribute to increased transmissibility. The intoduction also emphasizes the concerns and discussions surrounding XBB.1.5, particularly on social media platforms like Twitter. Recognizing the limitations of analyzing COVID-19 information on Twitter, the study aims to employ Social Network Analysis (SNA) to investigate the social dynamics, influencers, topics, and sentiments related to XBB.1.5 on social media. The study questions presented focus on social network structures, key influencers, frequent discussion issues, and expressed sentiments regarding XBB.1.5.

## Research methods

### Data collection

To investigate Twitter trends, the keywords, “XBB.1.5″, were used to perform the social network analysis method. NodeXL, a network analysis and visualization tool for Microsoft Excel, was used to perform SNA. NodeXL collects publicly available social media data and imports it into Microsoft Excel for analysis [20]. Some previous studies used SNA with nodeXl and Twitter as social media [21], [22]. In this study, NodeXL was implemented for data collection from Twitter [23] from December 23rd, 2022 to January 23rd, 2023. This tool used developer Twitter accounts to obtain metadata (e.g., date, time, number of retweets, and likes) and user information, as well as provided tweet IDs as edges. In MS Excel 2019, data collection was also performed on Microsoft Windows 11, by using NodeXL professional version 1.0.1.508.

### Data cleaning

The data obtained were also cleaned to eliminate duplication and irrelevant tweets, such as tweets that do not contain any information related to XBB.1.5. It includes removing duplicate tweets, filtering irrelevant content like spam or unrelated tweets, handling retweets appropriately, managing missing data, removing noise and irrelevant characters, and standardizing the data format. By performing data cleaning, researchers can ensure reliable and accurate results when analyzing the discourse on the XBB.1.5 variant on Twitter using NodeXL. Stopwords, punctuation, and special characters were also removed from the text of the tweets. Moreover, the user information and metadata were used to develop a user-tweet network, where content creators and tweets were nodes and tweets edges, respectively [24].

### Data analysis

This study used social network analysis (SNA) to examine Twitter communication data about XBB.1.5. The metrics of this network analysis was also used to identify top domains/sources, words, word pairs, mentions, tweeters (influencers), sentiment-related terms (positive, negative, or neutral), and centrality measures (degree, betweenness, and eigenvector centrality) [25]. In a network, the degree of a node often measures the number of connections possessed [26], and is divided into two parts, namely in-degree and out-degree. From this context, in-degree and out-degree are the numbers of incoming and outgoing connections to and from a node, respectively [27]. In-degree centrality especially depict the number of mentions or retweets received by a vertex and is also an essential metric in measuring social network [28]. Therefore, this study used degree centrality to identify the users actively participating in the XBB.1.5. discourse on social media, by having many followers. The betweenness centrality measure is the number of times a node serves as a bridge between other network nodes. This explains that the nodes with high betweenness have a central role in connecting different network parts [27]. In this study, betweenness centrality was subsequently employed to identify the users playing a central role in connecting different groups or communities within the XBB.1.5. discourse on social media.

Based on applicable principles, eigenvector centrality often explain that a node is considered important when connected to other essential nodes. This measure assigns a score to each node regarding the centrality of its neighbours. From this context, the node with high eigenvector centrality commonly has many connections to other branches with multiple links [27]. Therefore, this study implemented eigenvector centrality to identify the users that are central to the XBB.1.5. discourse on social media, due to their connection to other influential users. These various measures were employed to identify the most influential nodes in the network while understanding the patterns of connectivity among the users discussing XBB.1.5. on social media. Sentiment analysis was also applied to the tweets to classify the opinion of XBB.1.5 as positive, negative, or neutral. From this context, the language used in the tweets was analyzed to understand the topic of discussion, information sources, and the support available to individuals evaluating XBB.1.5 on Twitter. The results obtained were also confirmed by using Azure Machine Learning to calculate sentiment scores on the media platform [29]. Moreover, the sentiment analysis was grouped into three categories, namely (1) Positive, where tweets expressed hope, gratitude, awareness, or appreciation to combat the COVID-19 pandemic, specifically on the new variant, XBB.1.5, (2) Negative, which emphasized the frustration, anger, fear, or disappointment tweets related to XBB.1.5, *and (3) Neutral, where several tweets were unidentified.*

### Data visualization

The results, including nodes and edges, were generated in Microsoft Excel and imported into a network visualization program known as Gephi software (Version 0.1.0) [30]. From this context, Twitter users and tweets were represented as nodes and edges in the display of the user-tweet network, respectively. The size of the nodes also represented the number of followers, with a Fruchterman-Reingold layout implemented to depict edge networks [31].

## Results

### Data collection

Data were obtained from the tweets on Twitter, which contained a specific keyword, “XBB.1.5″. In this case, the total posts obtained globally were n = 43,394 tweets, retweets, and replies from 23rd December 2022 to 23rd January 2023. Table 1 presents the graph metrics before and after the data cleaning process. The graph represents the social network under investigation. The metrics measured include the number of vertices (representing individual users) and the total number of edges (representing connections between users) in the network. Before data deletion, the graph had a total of 29,111 vertices, indicating the initial number of users included in the analysis. Additionally, there were 71,623 edges, representing the connections between these users. After the data cleaning process, the number of vertices remained the same at 29,111, indicating that all the users originally included were retained in the cleaned dataset. However, the total number of edges decreased to 43,394 after the deletion of certain connections during the cleaning process. The decrease in the number of edges after data deletion suggests that some connections were deemed irrelevant, redundant, or noisy and were therefore removed to ensure the accuracy and quality of the network analysis.

**Table 1:** Graph Metrics Before and After Cleaning Data.

**Graph metrics**	**Before Deletion**	**After Deletion**
Vertices	29,111	29,111
Total edges	71,623	43,394

### Social network analysis (SNA)

**Fig. 1:**
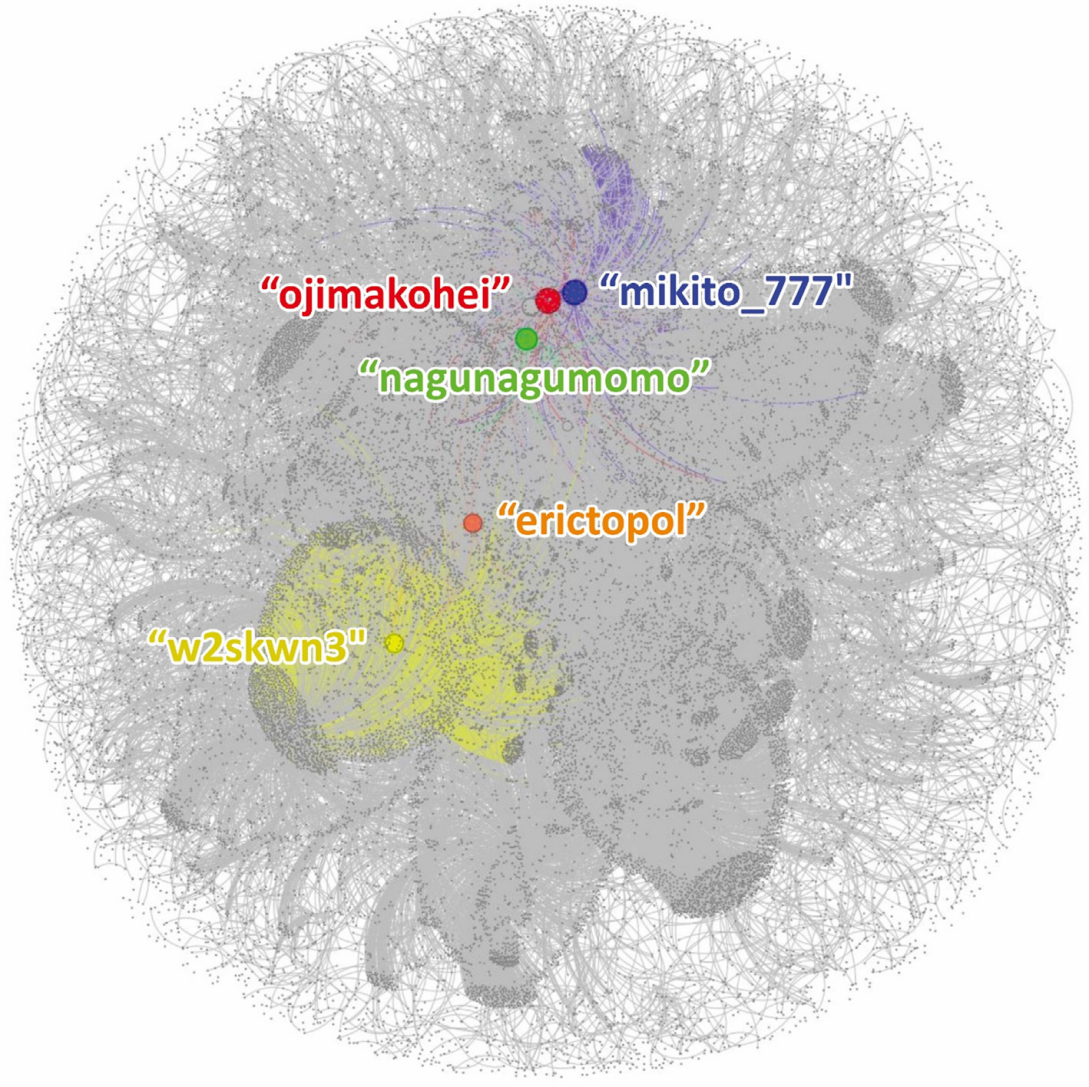
Social network graph of XBB.1.5. on Twitter.

Fig. 1 shows the Twitter users in social network graph clusters. This provides an overview of the network and key user groups tweeting about “XBB.1.5”. In this case, the media users and each line between them were represented by nodes and an edge, respectively. Based on Fig. 1, five key users were highlighted with different size circles and colours, based on the betweenness centrality score (BCS) explaining the rank of the node sizes. The effect of a vertex was also quantified on the flow of information between all other vertices, assuming data were distributed through the shortest pathways [32]. This proved that Twitter users were more influential, with each circle line emphasizing strong relationships with other media personalities.

In Fig. 1, the following Twitter users had the highest betweenness centrality score, namely “ojimakohei”(red), mikito_777 (blue), “nagunagumomo” (green), “erictopol” (orange), w2skwn3 (yellow). These were the influential Twitter users actively sharing information about XBB.1.5 and connecting with other personalities on the media platform. The users with the highest betweenness centrality scores were also mostly Japanese accounts, implying that the XBB.1.5 variant was highly evaluated in the country.

### Patterns or trends on XBB.1.5. Tweets

Table 2 shows the in-degree, out-degree, betweenness, closeness, and eigenvector centrality scores of the top 10 Twitter users to explain various patterns and trends. This proved that “ojimakohei” was highly central in the network, due to being among the top 10 users in all five measurements. Other common users in multiple measurements included “mikito_777″ and ”yfuruse“, regarding the possession of a significant role in the network. From these results, the centrality measurements prioritized the significance of the users, based on their network influence level and the ability to connect with other personalities.

**Table 2:** The top 10 Twitter users with the highest score of degree and centrality.

**Degree Measurement**		**Centrality Measurement**		
**In-Degree**	**Out-Degree**	**Betweenness Centrality**	**Closeness Centrality**	**Eigenvector Centrality**
mikito_777	zypisfy	ojimakohei	ojimakohei	mikito_777
ojimakohei	grutterstephan	mikito_777	erictopol	ojimakohei
nagunagumomo	nono_warrior	nagunagumomo	qbk_jitensya	yfuruse
chuckcallesto	ejustin46	erictopol	oribeyumi	bb45_colorado
w2skwn3	5wvcdvfcyrnczju	w2skwn3	5wvcdvfcyrnczju	5wvcdvfcyrnczju
bb45_colorado	haruo252525	tryangregory	ams_129	otakutokkai
erictopol	tryangregory	yfuruse	eumi_painthobby	ssn788_colorado
yfuruse	clannad992	rajlabn	triangle24	yujiy0402
ssn788_colorado	ping_jesus	triangle24	mikito_777	mindgaterz_2010
triangle24	qbk_jitensya	oribeyumi	haruo252525	triangle24

The patterns and trends of conversation were also identified by top URLs assessed within tweets. Table 3 provides an overview of the top 10 key URLs within tweets. These implemented links emphasized legitimate and high-quality information sources, such as peer-reviewed papers.

**Table 3:** Overview of the 10 most shared URLs.

**Top URLs in Tweet in Entire Graph**	**n**
https://news.yahoo.co.jp/articles/5e8b41fffc7cad69a8aaaaf1f89334894543c1a7	1166
https://www.biorxiv.org/content/10.1101/2023.01.22.525079v1	384
https://www3.nhk.or.jp/news/html/20230123/k10013957831000.html	261
https://www.politico.com/news/2023/01/25/bivalent-covid-booster-xbb-1-5-00079451	196
https://www.dailymail.co.uk/health/article-11677059/CDC-study-finds-bivalent-Covid-vaccines-provide-strong-protection-against-XBB-1-5-variant.html	182
https://www.cdc.gov/mmwr/volumes/72/wr/mm7205e1.htm?s_cid=mm7205e1_w	180
https://nordot.app/991104270561165312?c=39550187727945729	176
https://Twitter.com/kyodo_official/status/1617820913390718977	176
https://www.bousai.metro.tokyo.lg.jp/_res/projects/default_project/_page_/001/022/937/20230126_08.pdf	156
https://Twitter.com/pokrath/status/1610214972948156419	110

Based on Table 3, the top URLs distributed within tweets were primarily related to the news articles and official statements prioritizing COVID-19. From this context, the most shared URL was a news article from Yahoo Japan (n = 1166). This was accompanied by a preprint study on bioRxiv (n = 384), a news article from NHK (n = 261), as well as publications from Politico (n = 196) and the Daily Mail (1 8 2). Several URLs from the Centers for Disease Control and Prevention (CDC) (n = 180) and various Twitter accounts were also among the top shared links, such as kyodo official (n = 176) and pokrath (n = 110). This indicated that the conversation in the entire graph primarily focused on COVID-19, as well as the various updates and developments related to the pandemic. In line with the URL from Bousai.metro.tokyo.lg.jp (n = 176), information was specifically provided about COVID-19 in Tokyo, Japan. Some people were also sharing tweets from official sources on their timelines, preliminarily emphasizing the present condition of the pandemic, including regular updates on the disease, information on new variants such as XBB.1.5, and the effectiveness of vaccines.

### Top 10 key influencers and source on XBB.1.5. Tweets

Based on the implementation of NodeXL, the key influencers and source tweets are shown in Table 4, Table 5, which explain the top 10 domains (source) in tweets, replied-to and mentioned users, tweeters, and hashtags related to XBB.1.5. Besides being identified, the impacts of these influencers were also prioritized on the spread of information and discussion about XBB.1.5.

**Table 4:** The top 10 tweeters, replied and mentioned users.

**Top Tweeters**	**n**	**Top Replied**	**n**	**Top Mentioned**	**n**
ja8yum	4,897,613	ssn788_colorado	280	cdcmmwr	160
kakusan_rt	4,318,475	ojimakohei	64	barouchlab	117
yoshiki7111	2,692,150	bigbaddenis	29	airisyc	111
simanekomama	2,618,213	ianhanomansing	19	ocpowers5599	111
myfavoritescene	2,340,286	mikito_777	18	mrowe1625	111
kompascom	2,274,737	sabrinamalhi	17	cdcgov	106
1125e	2,215,513	txtdrpemerintah	16	who	66
detikcom	2,163,205	medical_for_all	16	cdcdirector	66
jurylady5	2,071,595	ryseto	14	fenitn	66
ecodaren	1,973,434	mikesington	14	enemyinastate	59

**Table 5:** Top 10 domains (source) and hashtags in tweets.

**Top Source**	**n**	**Top Hashtags**	**n**
Twitter.com	1425	covid19	451
co.jp	1365	omicron	180
or.jp	462	xbb15	172
biorxiv.org	459	xbb	172
cdc.gov	343	โควิด *	126
politico.com	217	bivalent	112
co.uk	207	mrna	111
nordot.app	186	sarscov2	106
lg.jp	181	コロナ5類移行に反対します **	92
tableau.com	122	covid	85

* Covid.

** I oppose the move to Corona 5.

According to Table 4, 'ja8yum' had the highest number of tweets (n = 4897613), accompanied by 'kakusan_rt' (n = 4318475) and 'yoshiki7111′ (n = 2692150). For replied users, 'ssn788_colorado' had the highest number of replies (n = 280), accompanied by 'ojimakohei' (n = 64) and 'bigbaddenis' (n = 29). The user, 'cdcmmwr', also had the greatest value for mentions (n = 160), accompanied by 'barouchlab' (n = 117) and 'airisyc' (n = 111). Moreover, the presence of news media (kompascom, detikcom) and health organizations (cdcmmwr, cdcgov, who, cdcdirector) were observed as top tweeters and mentioned, respectively. From these results, the key influencers or popular information sources related to XBB.1.5 were sequentially individual Twitter accounts, health organizations, and news media.

According to Table 5, tweets primarily originated from various sources such as Twitter, with a significant value emerging from Japanese outlets, namely co.jp and or.jp. A notable number of tweets also emerged from scientific sources, including biorxiv.org and cdc.gov. In this case, the top hashtags used were related to COVID-19 and the XBB.1.5 variant, such as #covid19, #xbb15, and #xbb. Moreover, the greatest hashtags in different languages were reported from Thailand, namely #โควิด (English: Covid), and Japan, including #コロナ5類移行に反対します(English: I oppose the move to Corona 5). Many tweets also evaluated COVID-19 generally, specifically the XBB.1.5 variant, indicating that the posts emerged from a mix of news media, health organizations, and individual users.

### Sentiment analysis

**Fig. 2:**
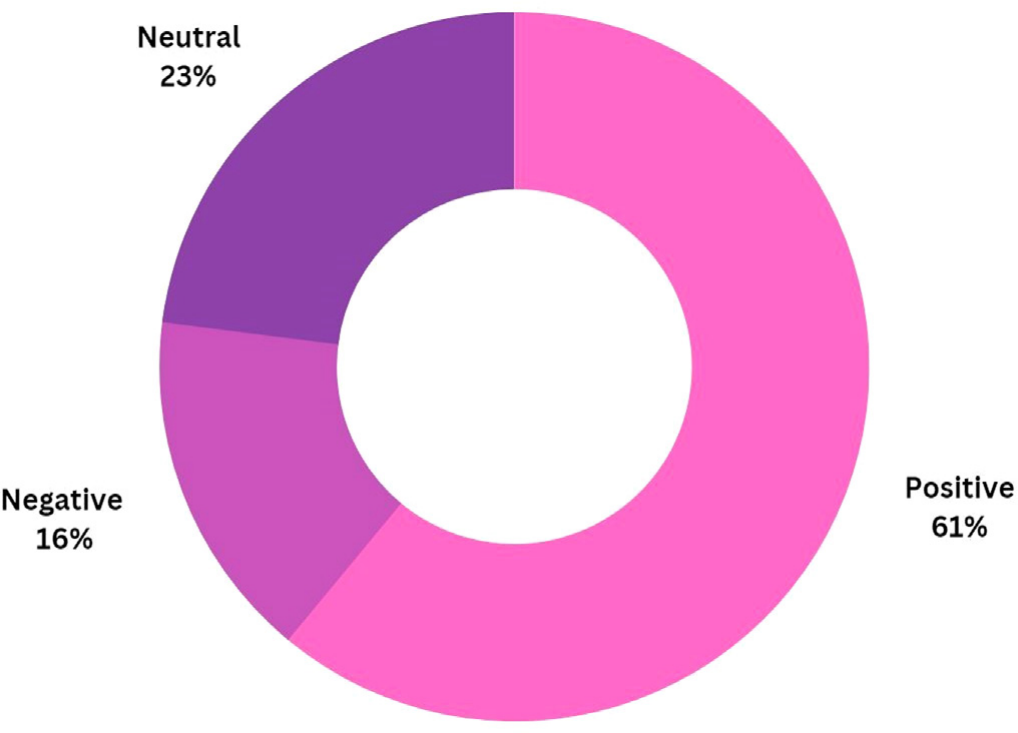
Sentiment analysis of retweeted posts.

Microsoft-based Azure Machine was used to measure the sentiment analysis, as explained in Fig. 2. This analysis indicated that the majority of the tweets (61.35 %) were positively classified. From this context, a significant portion of the tweets expressed an emphatic awareness opinion or emotion towards XBB 1.5. This was accompanied by a neutral and negative sentiment of 22.44 % and 16.20 %, respectively, indicating that Twitter users had no opinions higher than those providing unfavourable perspectives.

## Discussion

This is the first study implementing social network analysis to explore people’s communication, behaviours, and sentiments, regarding the new COVID-19 variant, omicron XBB.1.5. Insights and comprehensive understandings of the discourse surrounding XBB.1.5 were also provided on Twitter by analyzing the sentiment of tweets, the most influential users and organizations, as well as the frequently implemented hashtags and sources [33]. Based on the results, the network structure of the discourse consisted of Twitter accounts mostly originating from Japan, implying that the XBB.1.5 variant was highly considered in the country. From this context, the high number of new COVID-19 cases in Japan caused the public to increase their alertness and seek subsequent information from Twitter. This was in line with a recent study, where most Japanese demanded to highly understand different types of hazards, including the COVID-19 pandemic, towards strengthening their preparedness [34]. Regarding the *COVID-19 Weekly Epidemiological Update* published by the World Health Organization on January 11, 2023, the highest numbers of new weekly cases were reported from Japan, with the approximate incidental value reaching 1 million. Despite this, the Omicron XBB.1.5 variant was still predominantly reported from the United States of America (82.8 %), accompanied by The UK (8.1 %) and Denmark (2.2 %) (World Health Organization, 2023). According to a previous study, the public's attitude towards COVID-19 was tightly associated with the rate of health literacy (Silva & Santos, 2021). From these results, various Japanese users had better e-health literacy, due to their curiosity and willingness to learn more about XBB.1.5 through Twitter interaction [35].

The analysis of centrality measurements also showed that doctors, politicians, and journalists were the most influential people in this discourse. This suggested that people were more likely to share tweets, retweets, and replies with credible users in their areas of work [36]. It also proved that these professions played a crucial role in building the XBB.1.5 discourse, through the provision of trusted and reliable information. Furthermore, the key influencers, namely the top 10 tweeters, repliers, and mentioners emphasized individual Twitter accounts. This was accompanied by the health organizations and news media accounts, which implied a wide-range user population contributing to the flow of information regarding XBB.1.5. In public health situations, the information from medical officials were sufficiently crucial to dominate and control the social media, as well as minimize the possibility for the community to share false information [37]. Based on the results, the most-shared URLs on Twitter were news articles and official statements about COVID-19. Several URLs from the CDC were also among the top shared links, suggesting that people preferred to share information from verified and official sources such as the CDC [37], [38]. Meanwhile, the URLs from other health organizations such as WHO, were considerably absent in the XBB.1.5 conversations. This was because the CDC articles provided summarized information, compared to the WHO publications written in long paragraphs. From these results, the patterns by which people share easy-to-understand URLs were emphasized. The tweets shared from official sources by various people also primarily focused on the present situation of COVID-19, including regular pandemic updates, information on new variants (XBB.1.5), and the effectiveness of vaccines [39].

According to the results, most of the top domains (sources) used in XBB.1.5 discourse originated from Twitter, Japanese websites (co.jp and or.jp), and scientific analysis links (biorxiv.org and cdc.gov). This suggested that scientists highly employed the social media platform in sharing and explaining new COVID-19 updates. In this context, scientific analyses highly impacted Twitter users than the general public (Marcec & Likic, 2022). This encouraged the consideration of the platform as one of the most popular methods of obtaining information. Twitter had also become a good location to share information due to its capability in allowing people broadcast data and speak to their audiences (Michela et al., 2022). Furthermore, the sentiment analysis of the XBB.1.5 discourse was implemented to better understand the patterns by which people expressed their feelings, views, and outlook regarding the topic [40]. This analysis indicated that the majority of the tweets (61.35 %) were positively classified, accompanied by neutral (22.44 %) and negative (16.20 %) sentiments. From this context, a significant amount of tweets conveyed positive opinions and implied the community’s attitude toward XBB. 1.5. This positive sentiment was then expressed by the individuals concerned about the new COVID-19 variant. It was also dedicated to spreading awareness and showing support for the community [41]. Moreover, positive sentences on tweets played an important role in cautiously informing the public about XBB.1.5 while remaining calm and prioritizing their health.

This study provides guidance for those wishing to communicate relevant XBB.1.5 knowledge and spread awareness of social responsibility. This study also addresses the development of a COVID variant, sheds information on policy and societal contributions. However, this study also has several limitations. Firstly, the findings may not be generalizable to other variants as the analysis was based solely on Twitter data related to the omicron XBB.1.5 variant. Secondly, the study's reliance on publicly available tweets on Twitter introduces potential bias and may not fully represent the broader discourse on COVID-19. Additionally, the sample is dominated by Twitter accounts from Japan, limiting the representation of perspectives from other regions. Finally, language and cultural factors were not fully accounted for, and the study solely relied on publicly available data, excluding private or protected tweets.

## Conclusions

This study demonstrated the value of social network analysis in examining people's communication, behaviours, and sentiments on Twitter, regarding the new COVID-19 variant, omicron XBB.1.5. Based on the results, Japan was the most active country discussing the variant on Twitter, suggesting that the high number of new COVID-19 cases increased public alertness and willingness to seek subsequent information. The importance of influential users such as doctors, politicians, and journalists, was also highlighted, accompanied by the preference for sharing URLs from verified and official sources, including the CDC. Furthermore, the positive sentiment expressed on Twitter, regarding XBB.1.5, indicated the community's commitment to spreading awareness and prioritizing their health. Health professionals and the government can arm themselves with the pertinent online narratives that the public may be facing by comprehending the social media X.B.B 1.5 landscape. It is hoped that the government would be able to implement policies that have been widely discussed by the public and required both during and after the Covid-19 pandemic. Academic and professional societies should continue their research on and implementation of social media strategy for X.B.B 1.5 with a focus on becoming the network's broadcast centers to guarantee that the accurate information is amplified and prevent panic in the public. This analysis should be in collaboration with Twitter influencers to overcome the misinformation related to COVID-19 and its variants.

## Ethical statement

Ethical approval was not required. This article does not contain any studies with human or animal trials. Twitter announced allows third parties developer Twitter accounts to use APIs (Application Programming Interfaces) such as NodeXL Pro, to retrieve and analyze public Twitter data [42]. No informed consent or ethical approval was required since publicly available tweets were analyzed using automated tools. Anonymity and confidentiality of Twitter users were ensured. No risks or ethical concerns were identified.

## Funding

This research received no specific grant from any funding agency in the public, commercial, or not-for-profit sectors.

## CRediT authorship contribution statement

**Ikhwan Yuda Kusuma:** Conceptualization, Methodology, Formal analysis, Software, Data curation, Validation, Writing – original draft. **Hening Pratiwi:** Data curation, Validation, Writing – original draft. **Shafa Fitri Khairunnisa:** Data curation, Validation, Writing – original draft. **Dian Ayu Eka Pitaloka:** Data curation, Validation, Writing – review & editing, Funding acquisition. **Arie Arizandi Kurnianto:** .

## Declaration of Competing Interest

The authors declare that they have no known competing financial interests or personal relationships that could have appeared to influence the work reported in this paper.

## Data Availability

No data was used for the research described in the article.
